# Radiation enhances the delivery of antisense oligonucleotides and improves chemo-radiation efficacy in brain tumor xenografts

**DOI:** 10.1038/s41417-021-00324-6

**Published:** 2021-04-14

**Authors:** Prakash Ambady, Yingjen Jeffrey Wu, Cymon N. Kersch, Joshua M. Walker, Samantha Holland, Leslie L. Muldoon, Edward A. Neuwelt

**Affiliations:** 1 Department of Neurology, Oregon Health and Sciences University, Portland, OR USA; 2 Department of Cell, Developmental and Cancer Biology, Oregon Health and Science University, Portland, OR USA; 3 Department of Radiation Medicine, Oregon Health and Science University, Portland, OR USA; 4 Department of Neurosurgery, Oregon Health and Science University, Portland, OR USA; 5 Department of Veterans Affairs Medical Center, Office of Research and Development, Portland, OR USA

**Keywords:** CNS cancer, CNS cancer

## Abstract

Overexpression of O^6^-methylguanine DNA methyltransferase (MGMT) contributes to resistance to chemo-radiation therapy (CRT) in brain tumors. We previously demonstrated that non-ablative radiation improved delivery of anti-MGMT morpholino oligonucleotides (AMONs) to reduce MGMT levels in subcutaneous tumor xenografts. We evaluate this approach to enhance CRT efficacy in rat brain tumor xenograft models. The impact of radiation on targeted delivery was evaluated using fluorescent oligonucleotides (f-ON). In vitro, f-ON was localized to clathrin-coated vesicles, endosomes, and lysosomes using confocal microscopy in T98G glioma cells. In vivo, fluorescence was detected in pre-radiated, but not non-radiated Long Evans (non-tumor bearing) rat brains. Cranial radiation (2 Gy) followed by AMONs (intravenous, 10.5 mg/kg) reduced MGMT expression by 50% in both orthotopic cerebellar D283 medulloblastoma and intracerebral H460 non-small cell lung carcinoma (NSCLC) xenograft models. To evaluate the efficacy, AMONs concurrent with CRT (2 Gy radiation plus oral 20 mg/kg temozolomide ×4 days) reduced tumor volumes in the medulloblastoma model (*p* = 0.012), and a similar trend was found in the NSCLC brain metastasis model. We provide proof of concept for the use of non-ablative radiation to guide and enhance the delivery of morpholino oligonucleotides into brain tumor xenograft models to reduce MGMT levels and improve CRT efficacy.

## Introduction

The addition of concurrent temozolomide to radiation therapy (chemo-radiotherapy; CRT) improves survival in patients with glioblastoma [1]. The cytotoxicity of temozolomide, an oral alkylating chemotherapy agent is primarily due to DNA methylation at the O^6^ position of guanine, triggering the DNA mismatch pathway leading to tumor cell apoptosis. O^6^-methylguanine DNA methyltransferase (MGMT) is a key DNA repair protein that can scavenge the methyl-groups from guanine and is associated with resistance to temozolomide [2–5]. MGMT is epigenetically inactivated by gene promoter methylation (methylated MGMT) in about 45% of patient-derived glioblastoma and MGMT gene methylation status remains the most significant predictive biomarker for better outcomes after CRT [6]. Thus, MGMT provides a promising therapeutic target for over half of glioblastoma patients that have un-methylated MGMT.

Morpholino antisense oligonucleotides are short synthetic nucleotide sequences that bind to complementary messenger RNA and block protein synthesis. They are specific, water-soluble, non-toxic, uncharged, and extremely stable under physiologic conditions, and are not cleaved by endogenous nucleases, thus providing an excellent platform for potential targeted therapies in oncology [7–9]. However, effective and targeted delivery of morpholino oligonucleotides to tumor sites is challenging, especially to the brain, where delivery of systemically administered agents across the neurovascular unit (NVU) remains a major hurdle [9–11]. We have previously reported the successful use of a non-ablative dose of ionizing radiation to prime human cancer cells to enhance the uptake of unmodified anti-MGMT morpholino oligonucleotides (AMONs) sequences to block MGMT mRNA translation [10]. In vitro, radiation-enhanced AMONs delivery decreased MGMT protein and significantly reduced human cancer cell proliferation index and cell viability in conjunction with temozolomide treatment [10]. In vivo, radiation enhanced the targeted delivery of a single intravenous dose of AMONs to achieve 50% MGMT knockdown after 3 days. MGMT silencing only occurred in radiation-primed tumor sites in the subcutaneous tumor model. Targeted gene expression knockdown using morpholino antisense is rapidly advancing and shows significant translational potential based on recent FDA approvals [12, 13].

Fractionated ionizing radiation is already an integral part of brain tumor therapy and is known to transiently increase NVU permeability at early time points after exposure in a dose-dependent manner [14–17]. In this study, we evaluate the use of clinically relevant non-ablative doses of radiation to guide and enhance the delivery of intravenously administered AMONs to brain tumor sites to silence MGMT and enhance the efficacy of CRT in orthotopic rat models of MGMT-positive human brain tumors. Targeted silencing of MGMT in pre-radiated tumor sites using our novel approach, without measurable MGMT silencing in non-radiated sites, may limit systemic toxicities associated with prior approaches to deplete MGMT in combination with temozolomide [18–20].

## Materials and methods

### Cell lines and reagents

Human T98G glioma, H460 non-small cell lung carcinoma (NSCLC), and D283 medulloblastoma cell lines were obtained from American type culture collection (ATCC; Rockville, MD, USA) and were used at early passage numbers. Cells were cultured in recommended culture medium supplemented with 10% fetal bovine serum and 1% streptomycin/penicillin in a 5% CO_2_ atmosphere at 37 C. A 3′-carboxyfluorescein labeled scrambled morpholino oligonucleotide (f-ON) was purchased from Gene Tools, LLC (Philomath, OR, USA). Three unmodified anti-MGMT specific morpholino oligonucleotide sequences were obtained from Gene Tools and dosed as previously described [10]. The in vivo dosage of f-ON and AMON (10.5 mg/kg) used in this study was calculated/converted from the manufacturer suggested in vitro effective dosage (15 µM) of AMONs (molecular weight 8 kDa) with the assumption that the average blood volume in rats is around 10 ml. Temozolomide for oral gavage (Merck & Co, New Jersey, USA) was purchased from Oregon Health and Science University (OHSU) pharmacy and diluted in sterile water to a concentration of 2.5 mg/ml. Rabbit anti-MGMT (#2739), poly-ADP polymerase (PARP; #9532), early endosome antigen 1 (EEA1; #3288), LAMP1 (#9091), p27 kip1(#2552), bcl-XL (#2762) and clathrin (#4796) antibodies was purchased from Cell Signaling Technology (Davers, MA, USA). Human mitochondrial antigen (HMT; ab 196611) was purchased from Abcam (Cambridge, MA) Mouse anti-tubulin antibody (T9026) was purchased from Sigma (St Louis, MO, USA).

### Western immunoblot

Cancer cells or tissues were harvested at specified time points for each experiment. Whole-cell lysates were isolated and subjected to western blotting analysis as previously described [10]. Quantification of the immunoblotting signal of individual proteins was performed using the UN-SCAN-IT Gel software (Silk Scientific, Inc., Orem, UT, USA).

### In vitro f-ON uptake

Cancer cells were exposed to radiation (2 Gy) or no radiation (controls). Cells were incubated with f-ON (15 µM) 1 day later and Texas red conjugated Wheat germ agglutinin (1:500) for 1 h at 37 C, then fixed in fresh 3.7% paraformaldehyde at room temperature for 15 min and washed with PBS. Membranes were not permeabilized. Immunohistochemistry was performed as described previously [21]. Images were collected the following day using a 60×/1.4 NA oil immersion objective on an inverted Zeiss LSM780 confocal microscope maintained by the OHSU Advanced Light Microscopy Core (Carl Zeiss, Oberkochen, German). Representative optical sections were collected using identical settings on the microscope and were analyzed using FIJI/ImageJ (NIH, Bethesda, MD, USA, https://fiji.sc) [22]. To further confirm this observation is dependent upon radiation dosing and incubation timing in D283 cells, we treated cells with 0, 0.5, 1, and 2 Gy radiation and 1 day later cultured with f-ON (15 µM) for 4 or 24 h. Cells were then washed to remove free f-ON and then imaged using Axio Observer inverted microscope (Zeiss, Gottingen, Germany).

### Brain irradiation

The care and use of animals were approved by the institutional animal care and use committee and were under the supervision of the department of comparative medicine at OHSU. Rats were anesthetized with dexmedetomidine (0.6 mg/kg IP) and ketamine (15 mg/kg) and confined in a lead container so only specific brain areas were irradiated while avoiding critical structures such as eyes and nasal cavity to minimize radiation treatment-related toxicity. A total dose of 2 Gy in tumor-bearing athymic rats or 5 Gy in normal Long Evan rats was randomly given in a single fraction using the RadSource RS2000 Irradiator at OHSU small animal research imaging core or the Versa HD (Elekta, Stockholm, Sweden) linear accelerator in the department of radiation oncology [10]. The same irradiators were used for all animals included in any particular experiment. The dose of radiation differed by tumor cell type and the optimal dose was determined in vitro for each cell line used. As previously described, non-ablative radiation is defined as radiation doses <8 Gy per fraction [11].

### Xenograft models

Female nude (*rnu/rnu*) rats (200–250 g) from the breeding colony maintained in the animal facility at OHSU were anesthetized with intraperitoneal ketamine (60 mg/kg) and diazepam (7.5 mg/kg). MGMT expressing cancer cells (10^6^ cells/10 μL, >90% viability) were injected via a 2-mm diameter hole drilled in the skull using a 27-gauge needle at a rate of 1 μL/min. H460 human NSCLC were implanted into the right caudate nucleus (bregma = 0; lateral 0.31 cm; vertical—0.65 cm) while the D283 medulloblastoma cells were injected into the cerebellum (bregma = 0; posterior = −1.1, vertical = −0.5) in a stereotactic frame (David Kopf Instruments; Tujunga, CA).

### Fluorescent oligonucleotide delivery

For assessment of f-ON delivery in normal rat brain, female Long Evan rats (200–250 g) were purchase from Charles River (Wilmington, MA). Long Evan rats received IV f-ON (10.5 mg/kg; IV) either alone (*n* = 3) or 1 day after 5 Gy whole-brain irradiation (*n* = 3). Delivery was also assessed in D283 medulloblastoma cerebellar xenografts 14 days after cerebellar implantation. Rats received f-ON alone (*n* = 2) or 1 day after 2 Gy brain irradiation (*n* = 3). Brains were harvested 1 day after f-ON administration and sections were stained for human mitochnondrial antigen (HMT) as a marker of human tumor cells and ionized calcium-binding adapter molecule 1 (IBA1) as a marker of human tumor cells and microglia with Hoechst nuclear counterstain.

### AMON delivery

The effect of radiation on AMON delivery to the brain was assessed in the D283 cerebellar tumor model (*n* = 14) 21 days after tumor implantation and the H460 NSCLC intracerebral xenograft model (*n* = 12) 5 days after tumor implantation. Tumor-bearing rats received a single dose of 2 Gy radiation followed in 1 day with AMONs (10.5 mg/kg; IV) randomly given to half of the animals. Tumor-bearing cerebellum or brain hemisphere were harvested 3 days later. Relative MGMT protein expression was measured by immunoblotting of brain lysates and semi-quantified by immunoblotting signal intensity after normalized with tubulin protein.

### Tumor treatment studies

The in vitro impact of AMONs on CRT efficacy was evaluated in the D283 cells. D283 cells were treated with 0 or 2 Gy radiation and 1 day later cultured with AMON (15 µM) or saline for 2 days. Cells were then treated with temozolomide (100 µg/ml). One day after temozolomide treatment, the whole-cell lysate was collected for immunoblotting analysis and cell viability was determined using WST-1 reagent (Abcam, Cambridge, MA, USA), following the manufacturer’s protocol. The effect of AMONs on CRT efficacy was evaluated in the D283 cerebellar tumor model (*n* = 14) beginning 21 days after tumor implantation and the H460 NSCLC intracerebral xenograft model (*n* = 12) 5 days after tumor implantation. Tumor-bearing rats received a single dose of 2 Gy radiation plus four doses of oral temozolomide (20 mg/kg × 4 days). AMONs (10.5 mg/kg; IV) were randomly administered 24 h after radiation to half (*n* = 7) of animals in each model. Rats were euthanized 7 days after the start of CRT. Brains were excised, fixed in 10% buffered formalin, and egg-gelatin embedded for vibratome sectioning at 100 µm in the coronal plane. Every fifth brain section was stained for HMT and then imaged at 35 µm pixel diameter on an Epson 1640XL flatbed scanner using Adobe Photoshop software. Tumor volume was assessed using NIH ImageJ software by one of the authors (LLM) blinded to treatments as previously described [23].

### Statistical analysis

For in vivo animal AMON delivery and tumor treatment studies, a sample size of *n* = 6 is required to achieve a power of 85%. All data are presented as mean ± SEM. The Student *t* test was used for the comparison of measurable variants between two groups. *P* < 0.05 was considered statistically significant (GraphPad Prism 6.0; Graph Pad Software).

## Results

### In vitro delivery of f-ON

**Fig. 1:**
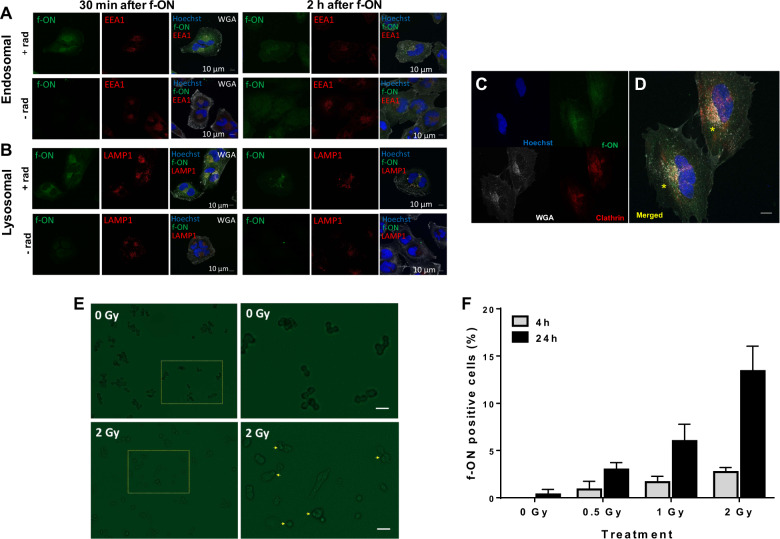
Non-ablative radiation enhanced in vitro uptake of fluorescein-labeled scrambled oligonucleotides (f-ON) into endosomes and lysosomes. T98G cells were treated with 2 Gy radiation (top rows) or no radiation (bottom rows) 1 day prior to the addition of f-ON (15 µM) for 30 min or 2 h. Cells were washed and then fixed and immunostained for EEA1 (endosome marker, (**A**) or LAMP1 (lysosome marker, (**B**) with a counterstain for nuclei (Hoechst) and cell membrane (WGA). **C**, **D** Intracellular f-ON co-localize with areas of strong clathrin expression in T98G cells (1-day post 2 Gy) after 30 min incubation with f-ON. **E**, **F** D283 cells were treated with 0, 0.5, 1, and 2 Gy radiation and 1 day later incubated with f-ON (15 µM) for 4 or 24 h. Cells were then washed to remove free f-AON and then imaged using Axio Observer inverted microscope (Zeiss, Gottingen, Germany). Bar scale = 15 µm. **E** Representative micrographs of cells given no-radiation (top row) and 2 Gy (bottom row) at 24 h after f-ON incubation, f-ON positive cells are indicated by the arrow. Micrographs in left and right panels were taken under 100X and 250X magnification, respectively. **F** Quantification of f-ON positive cells as a percentage. Data were presented as mean ± SD of six randomly selected fields of view. Rad radiation, EEA early endosome antigen, LAMP lysosome-associated membrane protein, WGA wheat germ agglutinin.

We assessed the impact of ionizing radiation (2 Gy) on the intracellular delivery of 3′-carboxyfluorescein labeled nonspecific f-ON administered 1 day after radiation in MGMT-expressing T98G glioma cells in vitro. Fluorescent signal was detected 30 min post f-ON incubation in radiated cells but not in non-radiated cells. Intracellular f-ON co-localized with both the endosome marker EEA1 (Fig. 1A) and lysosome-associate membrane protein 1 (LAMP1, Fig. 1B) at the 30 min time point. At 2 h after f-ON administration, the endosomal fluorescence was no longer detected but the lysosomal localization remained strong (Fig. 1A, B). Intracellular f-ON co-localized with areas of strong clathrin expression (Fig. 1C, D). We confirmed radiation dose and time-dependent uptake of f-ON in D283 medulloblastoma cells (Fig. 1E, F). Compared to non-radiated control, 24 h f-ON incubation in combination with 2 Gy radiation showed the most f-ON positive cells, with 13 ± 2% of cells showing positive cell-associated fluorescent signal. These observations support our previous finding that radiation activated the endocytosis trafficking pathway in a dose-dependent manner to enhance morpholino intracellular delivery.

### In vivo delivery of f-ON across the NVU

**Fig. 2:**
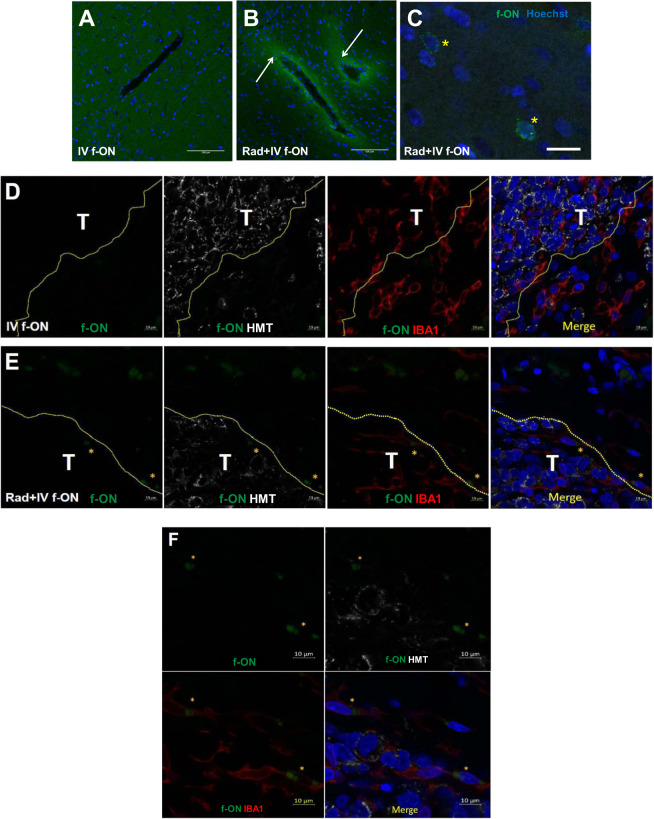
Non-ablative radiation enhanced the delivery of f-ON into rat brain in vivo. **A**–**C** Long Evan rats received IV f-ON (10.5 mg/kg; IV) either alone (**A**) or 1 day after 5 Gy whole-brain irradiation (**B**), (**C**). Brains were harvested 1 day after f-ON administration. Sections were counterstained with Hoechst nuclear stain and fluorescein localization was analyzed. **C** High magnification field showing punctate cellular uptake of f-ON. **D**–**F** Athymic nude rats with D283 tumor received f-ON alone (**D**) or 1 day after 2 Gy brain irradiation observed; representative micrographs under low magnification (**E**) or high magnification (**F**). Brains were harvested 1 day after f-ON administration and sections were stained for human mitochnondrial antigen (HMT) and ionized calcium-binding adapter molecule 1 (IBA1) as a marker of human tumor cells and microglia. Scale bar = 100 μm (**A**, **B**), 20 µm (**C**), or 10 µm (**D**, **E**, **F**), respectively. (T tumor and *representative f-ON positive cells).

We assessed the impact of cranial irradiation on the localization of f-ON in the brain in non-tumor-bearing Long Evans rats and tumor-bearing athymic nude rats. No fluorescent signal was found in rat brains treated with either IV f-ON alone without radiation (Fig. 2A). Non-tumor-bearing rats that received IV f-ON after radiation showed increased perivascular fluorescein signal in the brain (Fig. 2B). Under high magnification, parenchymal cells showed intracellular punctate fluorescence, suggesting the f-ON was taken up into endosome and lysosome cellular compartments (Fig. 2C). In the absence of radiation, rats bearing cerebellar D283 medulloblastoma xenografts showed minimal f-ON fluorescent signal that was limited to the necrotic regions within the tumor and was not found in the brain around the tumor (Fig. 2D). Fluorescent signal was increased in the irradiated tumors (Fig. 2E). Strong f-ON fluorescence was found predominantly but not limited to IBA1 positive cells in the brain around the tumor (Fig. 2F and Supplemental video). In addition, a diffuse parenchymal f-ON signal was detected as far as 200 µm from the tumor edge (Data not shown).

### Decreased MGMT by AMON in rat brain tumor xenografts

**Fig. 3:**
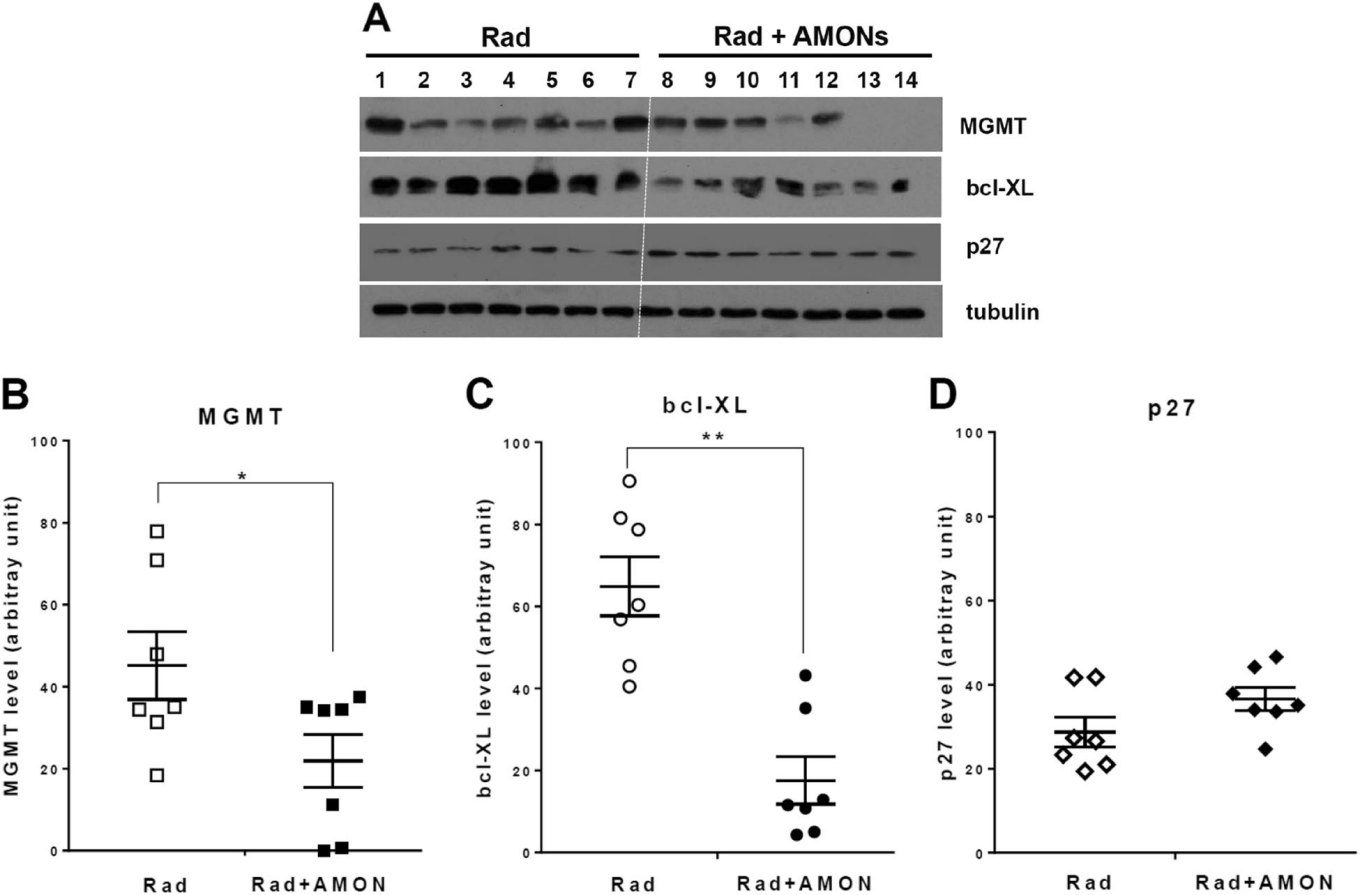
Radiation-guided delivery of anti-MGMT oligonucleotides (AMONs) to reduce MGMT protein expression in D283 medulloblastoma orthotropic brain tumor model. Athymic nude rats were inoculated with MGMT expressing D283 medulloblastoma cells (intra-cerebellar, *n* = 14). Rats received a single dose of 2 Gy cranial irradiation. AMON (10.5 mg/kg; IV) was administered 1 day after radiation to half (*n* = 7) of animals and all tumors were harvested 3 days later. **A** Immunoblot of MGMT, bcl-XL (apoptosis marker), and p27 (cell-cycle biomarker to confirm radiation delivery) of rat cerebellar tumors; Tubulin level was used as a total protein loading control. **B**–**D** Semi-quantification of MGMT, bcl-XL, and p27 immunoblotting signals. Data were presented as mean ± SEM. Statistical significance indicated by *P < 0.05. **P < 0.01.

In the D283 medulloblastoma orthotopic xenograft model, cranial radiation followed by IV AMON significantly decreased MGMT protein expression compared to radiation only (45.2 ± 8.2 vs. 21.9 ± 6.5 arbitrary unit, *p* = 0.04; Fig. 3A, B). Compared to radiation alone, radiation plus AMONS led to a significant reduction (64.9 ± 7.2 vs. 17.6 ± 5.8, *p* < 0.01) in the anti-apoptotic protein bcl-XL at 3 days (Fig. 3C). There was no difference in expression of p27, a biomarker used to confirm radiation exposure between these two groups (Fig. 3D). We found a non-statistically significant (*p* > 0.05) decrease in MGMT expression (35.7 ± 16.1 vs. 13.3 ± 8.2; Supplemental Fig. 1A, B) and bcl-XL (41.3 ± 9.3 vs. 23.3 ± 5.8; Supplemental Fig. 1C) expression comparing radiation alone to radiation plus AMON in H460 NSCLC xenografts (Supplemental Fig. 1).

### AMON enhances CRT efficacy in vitro of D283 cells and in MGMT expressing rat brain tumor xenografts

**Fig. 4:**
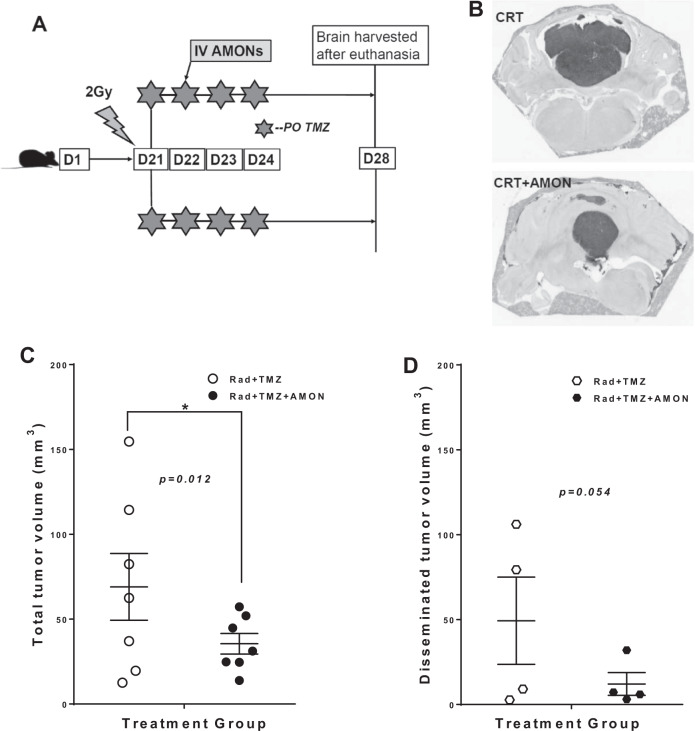
AMON increased the efficacy of chemo-radiation therapy (CRT) in the D283 medulloblastoma orthotropic brain tumor model. Athymic nude rats were inoculated with MGMT expressing D283 medulloblastoma cerebellar orthotropic xenografts (*n* = 14). Tumor-bearing rats received a single dose of 2 Gy radiation with four daily doses of oral temozolomide (20 mg/kg × 4 days). AMONs (10.5 mg/kg; IV) were administered 1 day after radiation to half (*n* = 7) of animals and all tumors were harvested 7 days after the start of CRT. **A** Scheme of experimental design and treatment regime. **B** Representative after-treatment brain tumor histology from each treatment group. **C** Total tumor volumes and (**D**) disseminated tumor volumes are shown for individual animals. Data were presented as mean ± SEM. Statistical significance indicated by *P < 0.05. **P < 0.01.

Compared to traditional CRT (2 Gy + temozolomide), we demonstrated the addition of AMONs (15 µM) at 24 h after 2 Gy radiation, significantly reduced D283 medulloblastoma cell viability (Supplemental Fig. 2A), and increase the presence of pro-apoptotic cleaved PARP protein (Supplemental Fig. 2B). These findings consistently agree with our previous publication. Furthermore, rats with orthotopic D283 medulloblastoma were treated with either CRT (radiation and temozolomide) or CRT with the addition of AMONs (experimental schema is shown in Fig. 4A). The optimal radiation dose and timing sequences were determined from our prior studies [10]. Tumor volume was 69.0 ± 19.7 mm^3^ after CRT only; addition of AMONs decreased tumor volume to 35.51 ± 6.08 mm^3^ (*p* = 0.012; Fig. 4B, C). Four of seven animals in each group (57%) showed disseminated leptomeningeal disease. In those animals that developed the leptomeningeal disease, AMON induced a 75% decrease of leptomeningeal tumor volume compared to CRT alone (12.1 ± 6.7 vs. 49.3 ± 25.7 mm^3^; Fig. 4D), but the *p*-value was not significant (*p* = 0.054) due to the small sample size (*n* = 4 per group). No significant difference in percentage body weight loss was reported between AMON and CRT treatment groups (3.8 ± 1.5% vs. 2.8 ± 1.2%), suggesting that there were no additional systemic toxic effects of IV AMON administration compared to CRT alone. The effect of AMONs was also tested in the H460 NSCLC brain metastasis model (Supplemental Fig. 3). Although not statistically significant, we found a similar trend toward decreased tumor volume with the addition of AMONs, compared to CRT alone (54.6 ± 32.4 vs. 91.4 ± 38.9 mm^3^, *p* = 0.48; Supplemental Fig. 3B, C).

## Discussion

The field of oligonucleotide therapeutics is rapidly evolving with the first antisense drug in the market and with promising agents in late-stage clinical trials [23–25]. Two major challenges in advancing oligonucleotide-based therapeutics for brain tumors including: (1) delivery of systemically administered oligonucleotides across the NVU and (2) convey the oligonucleotides to the correct intracellular compartment to achieve the desired effect within tumor tissues [11, 26, 27]. Unmodified morpholino oligonucleotides have very limited in vivo or in vitro ability to efficiently penetrate the eukaryotic plasma membrane without the aid of delivery strategies such as endo-porter or structural modifications [28]. The AMONs used in this study have 21–25 base pairs (molecular weight 7–9 kDa) and are expected to have very limited passive permeability across the NVU, even in enhancing brain tumors [29, 30]. Prior studies in rodent models have attempted to overcome the barrier of the NVU by using intra-ventricular or direct interstitial micro-perfusion of morpholinos to silence various target proteins in the CNS [31–33]. Drug administered into the cerebrospinal fluid is rapidly cleared into the blood via bulk flow through the CSF [34], while drug penetration from the CSF into brain parenchyma is poor and can range from as little as 0.3 mm for biologically active substances such as brain-derived neurotrophic factor to 2 mm for inert small molecules like hydroxyurea [35, 36]. We have previously demonstrated the novel use of a non-ablative dose of radiation in combination with IV AMONs to achieve MGMT silencing in radiation primed tumors in a subcutaneous tumor model [10]. We have previously hypothesized that radiation may enhance the physiological dynamin-mediated endocytosis as demonstrated by the upregulation of caveolin 1 at 30 min after radiation, while clathrin and dynamin2 expression peaked at 24–48 h after ionizing radiation. We adopted the radiation dose (2 Gy) and timing (24 h) of AMON administration based on our prior publication [10]. Here (Fig. 1E, F), we further demonstrate the best intracellular delivery of f-ONs when D283 cells were given 24 h after 2 Gy radiation. Confocal fractionated cranial radiation (1.8–2 Gy) delivered over 6 weeks is the standard-of-care treatment for glioblastomas and other primary brain tumors including medulloblastoma [1]. Thus the choice of 2 Gy for our experiments provides additional clinical and translational significance.

Preclinical and clinical evidence suggests that in addition to direct DNA damage, radiation therapy can induce transient focal NVU disruption [37, 38]. We found that radiation enhanced the delivery of f-ON across the NVU in normal and tumor-bearing rats, and radiation-induced physiologic endocytosis plays a key role in the intracellular delivery of morpholinos. MGMT was used as a prototypic protein and temozolomide as chemotherapy in this proof of concept study. We found that radiation-enhanced delivery of AMONs blocks MGMT synthesis by interfering with the translation of MGMT mRNA and specifically decreased MGMT expression in intracerebral tumors. Thus, the use of carefully contoured delivery of non-ablative radiation dose fractions holds the translational potential to enhance the delivery of AMONs in the targeted field without significant uptake in non-radiated sites. Radiation exposure is well known to enhance the expression of key proteins in the physiological endocytosis pathway, possibly mediating intracellular delivery of morpholino oligomers. This study provides additional evidence to support the hypothesis that once they cross the NVU, morpholino oligomers are taken up into the intracellular compartment through the endocytic pathway as evident by fluorescent signal localized to clathrin-coated vesicles, endosomes, and lysosomes using confocal microscopy (Fig. 1). Further, we demonstrate in vivo fluorescent signal in pre-radiated non-tumor bearing rat brain after intravenous administration of f-ONs but not in non-radiated rat brains (Fig. 2). Our observations suggest that radiation-enhanced delivery may be beneficial in silencing target proteins in both radiologically non-enhancing as well as enhancing brain tumors within the contours of the radiation field. Targeted radiation-guided delivery limits off-target pharmacodynamic effects, bone marrow, and other systemic toxicities frequently seen with alternative approaches using MGMT pseudo-substrates such as O^6^-benzylguanine and O^6^-(4-bromothenyl) guanine [39–41]. No significant difference in percentage body weight loss was reported between AMON and CRT treatment groups in our study.

The role of MGMT is most relevant in the management of glioblastoma. One major limitation of the current studies is the lack of MGMT-expressing glioblastoma xenograft models. We have tested multiple commercially available and patient-derived un-methylated MGMT glioblastoma lines from our collaborators but failed to form MGMT protein-expressing brain tumors after orthotropic inoculation in athymic rats (data not shown). We hypothesize that this may reflect an epigenetic change in the in vivo tumor methylation status after implantation. Further, it is well known that there is a discordance between *MGMT* methylation as measured by methylation-specific polymerase chain reaction and *MGMT* mRNA levels and protein expression [42]. Hence, we used established H460 NSCLC and D283 medulloblastoma xenografts that continue to express MGMT in nude rat brains. Both cancer cell lines are traditionally resistant to alkylating agents potentially due to high levels of MGMT protein expression. In contrast to D283, H460 cells are more resistant to radiation. In addition, we also noted a large intertumoral heterogeneity in tumor volumes, this may have impacted the lack of statistical significance in our H460 model, even though we noted a strong trend toward decreased MGMT and increased CRT efficacy by AMONs. The target protein silencing achieved with morpholino oligomers is transient (3–7 days) [11]. However, the current standard of care using daily dose (2 Gy) fractions of radiation over 6 weeks provides a window of opportunity for a potential daily AMON dosing schedule and sustained MGMT silencing over this time period to enhance the efficacy of CRT in un-methylated for high-grade glioma and potentially other primary brain tumors such as medulloblastoma [2, 43].

In conclusion, we demonstrated that compared to CRT alone, the addition of a single dose IV AMON combined with CRT significantly enhanced CRT efficacy in an MGMT expressing xenograft brain tumor model. We provide hypothesis-generating data to support further evaluation of IV AMONs in combination with standard CRT regimes to enhance its efficacy in MGMT overexpressing brain tumors. The dosage, frequency, and timing of AMON and optimal radiation dose fractions to achieve optimal efficacy needs further investigated and defined in future small animal and early phases in human studies. This study also supports further exploration of our approach to silence alternate clinically relevant target cancer-related proteins to enhance the efficacy of CRT.

## Supplementary information

Supplemental data

Supplementary video
