# Supplementary material for: Radiation enhances the delivery of antisense oligonucleotides and improves chemo-radiation efficacy in brain tumor xenografts
Source: Cancer Gene Ther. 2021 Apr 14;29(5):533–42. doi: 10.1038/s41417-021-00324-6 (PMC9113935; doi:10.1038/s41417-021-00324-6)
Supplement: Supplementary file 1 — Supplemental data [file 41417_2021_324_MOESM1_ESM.docx]

**Supplemental data:**

**Supplemental Video 1. (z-stack images) of athymic rat brain treated with radiation and IV f-ON *in vivo*.** Athymic nude rats with D283 tumor received f-ON 1 d after 2 Gy brain irradiation. Brains were harvested 1 d after f-ON administration and sections were stained for human mitochnondrial antigen (HMT) and ionized calcium-binding adapter molecule 1 (IBA1) as a marker of human tumor cells and microglia. Scale bar =10μm

**Supplemental Figure 1.** **Radiation enhanced AMON delivery and tumoral MGMT protein expression in H460** **NSCLC** **brain tumor xenografts.** Athymic nude rats were inoculated with MGMT expressing H460 NSCLC intra-cerebrally (n=12); A single dose of 2 Gy radiation was given at right hemisphere tumor area. AMONs (10.5 mg/kg; IV) were administered 1 d after radiation to half (n=6) of animals and all tumors were harvested 3 d later; **A**) Immunoblot of MGMT, bcl-XL and p27 of rat cerebral tumors; Tubulin level was used as total protein loading control. Reduction in MGMT expression was found only in tumors that received both radiation and AMONs; **B**) Semi-quantification of MGMT, bcl-XL and p27 immunobloting signals. Data were presented as mean ± SEM.

**Supplemental Figure 2. Radiation enhanced delivery of AMON enhances the in-vitro efficacy of chemo-radiation.** D283 cells were treated with either 0 or 2 Gy radiation and 1 d later cultured with AMON (15 µM) or saline 2 days. Cells were then treated with TMZ (100 µg/ml) and cell viability was measured by A) wst-1 assay or B) immunoblot analysis at 24 hr after TMZ. CRT= 2 Gy + TMZ. Relative cell viability (%) was calculated by normalizing with mean of untreated control. Data were presented as mean ± SD of six individual wells of each treatment group. Apoptosis is indicated by the presence of cleaved PARP protein. * indicates p<0.05.

**Supplemental Figure 3. Addition of AMON and the chemo-radiation therapy (CRT) efficacy in H460 NSCLC** **brain tumor xenografts.** Athymic nude rats were inoculated with MGMT expressing H460 NSCLC intra-cerebrally (n=12); A single dose of 2 Gy radiation + oral temozolomide (20 mg/kg x 4 d) as standard chemo-radiation therapy (CRT). AMONs (10.5 mg/kg; IV) were administered 24 h after radiation to half (n=6) of animals and all tumors were harvested 7 d after start of CRT; **A**) total tumor volume and **B**) representative brain tumor histology from each treatment group. There is 40% reduction of total tumor volume in animals that received both AMON and CRT; Data were presented as mean ± SEM.
